# Pharmacokinetics of anidulafungin during venovenous extracorporeal membrane oxygenation

**DOI:** 10.1186/s13054-016-1501-4

**Published:** 2016-10-17

**Authors:** Gerardo Aguilar, Rafael Ferriols, José A. Carbonell, Carlos Ezquer, José Miguel Alonso, Abigail Villena, Jaume Puig, David Navarro, Manuel Alós, F. Javier Belda

**Affiliations:** 1 Department of Anesthesiology and Intensive Care, Surgical Intensive Care Unit, Hospital Clínico Universitario de Valencia, Avenida Blasco Ibáñez, 17, 46010 Valencia, Spain; 2 Department of Pharmacy, Hospital Clínico Universitario de Valencia, Avenida Blasco Ibáñez, 17, 46010 Valencia, Spain; 3 INCLIVA, Institute of Research, Avenida Blasco Ibáñez, 17, 46010 Valencia, Spain; 4 Department of Microbiology, Hospital Clínico Universitario de Valencia, Avenida Blasco Ibáñez, 17, 46010 Valencia, Spain; 5 School of Medicine, University of Valencia, Avenida Blasco Ibáñez, 15, 46010 Valencia, Spain

**Keywords:** Echinocandins, Extracorporeal membrane oxygenation, Acute respiratory distress syndrome

Echinocandins are currently considered the first-line treatment for invasive candidiasis (IC) in the intensive care unit (ICU) [1, 2]. However, extracorporeal membrane oxygenation (ECMO), a rescue therapy used in patients with severe acute respiratory distress syndrome (ARDS) [3], could alter the pharmacokinetics of certain drugs [4]. We prescribed anidulafungin for suspected IC in a patient with severe ARDS on ECMO and measured the plasma concentrations of the drug using high-performance liquid chromatography (HPLC).

A 69-year-old male patient was admitted to the ICU with septic shock secondary to peritonitis. The anti-infective treatment was based on surgical source control and broad spectrum antimicrobial therapy, including anidulafungin at usual doses. The patient developed severe ARDS. ECMO with Novalung iLA Activve™ was initiated, maintaining ultraprotective ventilation. Femoral (23 F) and jugular (19 F) cannulas (Novalung™, Germany) were inserted with 4.5 L/min blood flow and 4 L/min gas flow. Urine samples and pre-filter and post-filter blood samples were collected before starting the eight-dose anidulafungin infusion and 0.5, 1, 1.5, 2, 4, 6, 8, and 24 h after the infusion ended. Anidulafungin was well tolerated without relevant adverse effects.

A non-compartmental pharmacokinetic analysis was performed using Abbottbase Pharmacokinetic Systems™ (Abbott Laboratories, Illinois, USA). The maximum and trough plasma concentrations (C_max_ and C_min_, respectively) were estimated directly from concentration-time data. The area under the plasma concentration-time curve over the 24-h dosing interval (AUC_0–24_) was estimated using the linear trapezoidal rule for both pre-filter and post-filter data. Clearance (CL) was estimated as dose/AUC_0–24._ The apparent volume of distribution at steady state (V_ss_) was estimated as the product of CL and mean residence time (MRT).

C_max_ and C_min_ were 13.5 and 2.19 mg/L, respectively (Fig. 1). Pre-filter and post-filter AUC_0–24_ were 107 and 111 mg/h/L, respectively; V_ss_ was 18.9 L; CL was 0.933 L/h. Urine anidulafungin concentrations were negligible. All pharmacokinetic data were comparable to published data in critically ill patients with and without other types of extracorporeal support [5].

**Fig. 1:**
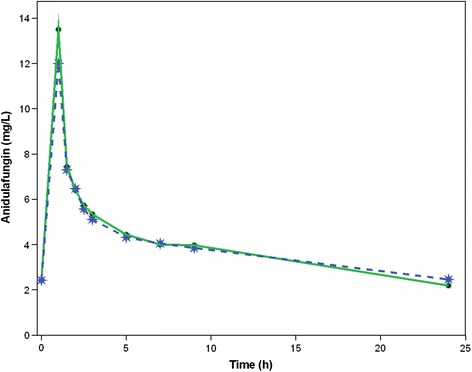
Plasma anidulafungin concentrations over 24 h. Solid and dotted lines represent the concentrations in the pre-filter and post-filter sides of the membrane, respectively. Urine concentrations of anidulafungin were very low and close to the limit of detection for the analytical procedure used (0.05 mg/L)

Regarding the use of anti-infective drugs in patients on ECMO, most pharmacokinetic data on this topic are from neonatal studies of antibiotics [4]. To the best of our knowledge, this report is the first on the pharmacokinetics of anidulafungin in a critically ill patient on ECMO. In our case, the therapy had little effect on the pharmacokinetics, suggesting that the dose of anidulafungin does not need adjustment. However, future studies are needed to confirm these findings.

## Abbreviations

ARDS: Acute respiratory distress syndrome
AUC_0–24_: Area under the plasma concentration-time curve over the 24-h dosing interval
CL: Clearance
C_max_: Maximum plasma concentration
C_min_: Trough plasma concentration
ECMO: Extracorporeal membrane oxygenation
HPLC: High-performance liquid chromatography
IC: Invasive candidiasis
ICU: Intensive care unit
MRT: Mean residence time
V_ss_: Apparent volume of distribution at steady state
